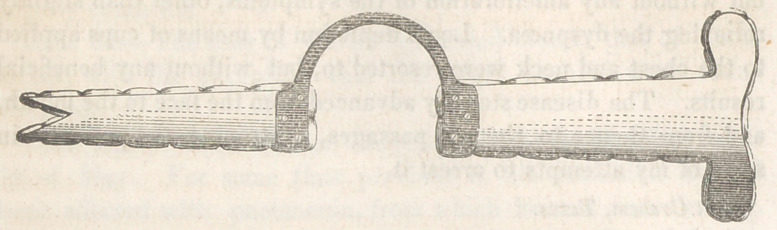# Surgical Cases

**Published:** 1849-07

**Authors:** J. M. Steiner

**Affiliations:** Assistant Surgeon U. S. Army; Fort Grahem, Texas


### MEDICAL EXAMINER,

AND

### RECORD OF MEDICAL SCIENCE.

NEW SERIES.—NO. LV.—JULY, 1849.

### ORIGINAL COMMUNICATIONS.

### *Surgical Cases, by* J. M. Steiner, M. D., *Assistant Surgeon U. S. Army.*

(Communicated in a letter to one of the Editors.)

TRAUMATIC TETANUS—CURE.

Corporal Wolf, of Comp. F., 2d Regt. U. S. Drgs., a native of Germany, of strong constitution and bilious temperament, aged 25, reported sick on the 1st of January. He complained of stiffness about the muscles of the throat and jaws, difficulty in swallowing, and an uneasy sensation about the scrobiculus cordis. He informed me that several days previous he had stepped on a thorn, which gave him so little uneasiness that he did not deem it necessary to report the accident. Upon examining the puncture near the base of the great toe, I could detect no evidences of inflammation. The pulse being strong and full, I bled him to the amount of sixteen ounces, and gave him $\frac{1}{4}$ gr. of the sulph. morph. At 3 P. M. he took v. gtts. of the oleum tiglii, after which $\frac{1}{4}$ gr. of morphia was administered every three hours during the night. On the morning of the 2d I found him much worse. The jaws were firmly closed, the muscles of the back of the neck hard and contracted, and he was unable to assume a sitting posture. Having sufficiently tested (when at Jalapa, Mexico, 1847) the treatment of traumatic tetanus by strychnia, so highly extolled by a writer in the New York Medical Journal, and found its effects to be null, I determined to put in practice a plan of treatment similar in some respects to that recommended by Dr. Hartshorne. I applied the actual cautery along the course of the spine, from the occiput to the sacrum, and dressed the surface with strips of lint, saturated with the tincture opii. At 11 A. M. I gave him viii. gtts. more of the oleum tiglii, as that given him the night previous had not yet operated. At 3 P. M. he was evidently much better, his bowels had been freely moved, and the distressing symptoms of rigidity about the jaws and neck considerably ameliorated. The tincture of opium was applied to the spine until the 9th of January when all the symptoms having disappeared, further treatment was dispensed with. There was no recurrence of the symptoms. As regards the treatment of this disease with strychnia, I am forced to report against it. I gave it a full trial at Jalapa after the battle of Cerro Gordo, where I tried it in five cases, but without the least benefit. These patients, however, were subject to other treatment at the same time. Opium was freely administered, injections of the infusion of tobacco, hot applications to the spine, &c. &c.

WOUND OF THE KNEE JOINT, FOLLOWED BY PHLEBITIS—CURE.

Augustus Helmering, of Company F., 2d. Dragoons, on the 23d of May, accidently punctured the left knee-joint with a knife. Ignorant of the extent of his injury, he did not report himself until the evening of the 24th, when he was taken into the hospital and his wound examined. I found the knee and the parts above and below for some inches red, inflamed, and very much swollen. The knife had entered upon the inner side of the patella inflicting a wound one inch in length, and extending into the joint. The edges or lips of the wound were erected and swollen, and the discharge consisted of synovia stained with blood. The knee to the touch was exceedingly painful. There was considerable constitutional fever; pulse strong and frequent; skin hot and dry, attended with complete loss of appetite and a great desire for cooling drinks. Mr. Amesberry's splint for fractured thigh was adjusted to the limb which was inclined upwards at an angle of 35, and the cold water dressing was ordered to be constantly applied. A draught of the sulphate of magnesia was given at 5 P. M., followed by an anodyne at 11 the same night.

On the morning of the 25th the local and constitutional symptoms were greatly aggravated. The whole extremity from the knee to the groin was greatly enlarged, and the pain was almost insupportable. As he complained of the cold water applications, I ordered them to be discontinued, and the warm water dressing of Mr. Liston substituted. The salts not having operated, the dose was repeated, and 15 ounces of blood taken from the arm. In the evening I found him better, the constitutional symptoms had abated in violence, and be begged me to allow the warm water dressing to be continued. As he had not been able to sleep the two previous nights I ordered him at three, P. M., 3 grs. of opium. On the 26th, upon visiting the hospital, I found the limb had increased in size, being greatly swollen from the foot to the groin. No pain of any consequence, but complained of feeling chilly. The warm water dressing was continued. In the evening his pulse was ninety, and a small quantity of pus could be detected in the discharge from the wound. In the night he took 3 grs. of opium. 27th. No fever, pulse more natural, slight restlessness, swelling of the extremity the same. The local treatment was continued and opium administered at bed time. 28th. I found him much worse. Constitutional symptoms had returned with double force. He was feverish, restless, with injected eyes, and complained of great pain in the left thigh. Upon examination I found that phlebitis had ensued, the femoral vein being inflamed as far as the groin, the course of which could be distinctly seen by a red line upon the surface. Upon pressure being made with the finger I could trace the vein, which felt hard like a cord. I ordered three dozen leeches to be applied to the thigh along the course of the inflamed vessel, and as the pulse was frequent and strong, bled him from the arm to the amount of sixteen ounces. The warm water dressing was continued, and opium freely administered. A diet of beef tea was allowed. In the evening he expressed himself greatly relieved.

29th. Febrile symptoms almost entirely disappeared, very little pain along the course of the femoral vein, complained much of weakness. Opened his bowels by injection, and continued warm water dressing. A diet of beef tea and soft eggs, with two or three glasses of wine allowed during the day.

30th. Pulse was frequent but not hard, red line along the course of the vein almost entirely disappeared, with the hardness upon pressure.

31st. The thigh felt soft and puffy, with the exception of the parts near the knee which were indurated and discolored; suffered no pain, but complained of having been troubled with diarrhœa during the night.

1st. June. The whole extremity looked much better; swelling had somewhat decreased, but the diarrhœa continued, the discharges becoming more frequent and copious. On the 4th of June the diarrhœa ceased without any medicines having been given to check it, other than the opium he had been accustomed to take at bedtime, without which he could obtain no rest. On the 7th the swelling from the knee to the groin had greatly decreased, but from the wound downwards there was no perceptible diminution. On the 8th he was placed in a litter to be conveyed to this place. From that time he rapidly improved without any bad symptoms intervening, and is at present, 30th, able to walk about with the assistance of a crutch. The wound is entirely closed, and the knee is not anchylosed. From a robust man he was reduced almost to a skeleton, but is now rapidly gaining flesh and strength. Throughout the whole progress of this man's illness the wound did not discharge more than two ounces of pus. There was at first a considerable discharge of synovia, with a little blood, which ceased in a few days. It will be seen that I gave no medicine to check the copious diarrhœa which commenced on the 30th, and ended on the 4th. I believed at the time, and I am now convinced, that it was an effort of nature to rid the system of the pus, which, produced by the phlebitis, mingled directly and unchanged with the venous blood, and if not thrown out of the system by some such recourse of nature, would have given rise to abscesses in the lungs or liver, and killed him. I have had during the war three other cases of phlebitis of the femoral vein supervening upon wounds of the lower extremities, all of which died; post mortem examination demonstrating the existence of abscesses in the lungs, which were undoubtedly the result of the phlebitis.

I have since had two cases of gun shot wounds followed by phlebitis, one of whom, a citizen of this state, died; the other, Moses H. McCullough, a soldier of F. Company, 2d Dragoons, of bilious temperament, and strong constitution, recovered—but what I consider of importance, resolution, as in the case of Helmering, was accompanied by profuse diarrhœa. From the foregoing cases would it not be rational treatment to address our remedies to the mucous surface of the intestinal canal, and endeavor to produce a determination to that membrane ?

ERYSIPELAS ATTACKING THE MUCOUS MEMBRANE OF THE LUNGS.

Five of the patients who died during the month were attacked after their admission into the hospital, with erysipelas, and its course and the peculiar effects it gave birth to were so unusual that I deem them worthy of being mentioned. Private Bulthaller was received into the hospital, and placed under treatment for epilepsy, with which he had been affected for several months. A few days after his admission he was attacked with erysipelas, the disease first appearing near the surface of the alæ nasi, and spreading thence rapidly over the face and scalp. On the third day after the appearance of the erysipelas, the mucous membrane of the mouth, tongue, and fauces, became affected, and that of the larynx, trachea and lungs became also involved. Upon ausculting the lungs, both rhoncus and silibus were distinctly audible, and the respiration became so difficult that he was in momentary danger of suffocation. On the sixth day, crepitation large and small could be detected by applying the ear to the chest. The same day he died, laboring under all the symptoms usually accompanying laryngitis and bronchitis in their acute forms. Upon examination of the lungs after death, the mucous membrane of the larger and smaller bronchi were found extensively inflamed, the chink of the glottis was nearly closed up by the effusion of serum beneath the lining membrane. The air cells and smaller bronchi were filled with a sero-purulent fluid.

Case 2d.—The case of Shoemaker was somewhat similar. He was affected in the first instance with symptoms of gastritis, which were disappearing under the treatment adopted, when, on the fourth day after his admission, erysipelas supervened. The course of the disease was much the same as that of Bulthaller, with the exception that the pulmonary apparatus was not so extensively involved. He died eventually of asphyxia. Upon examining the larynx, the rima and epiglottis were found preternaturally swollen, accompanied by an effusion of serum beneath the mucous membrane, which was highly inflamed, and in some places slightly eroded. There was very little liquid found within the lungs, and the mucous membrane of the bronchi and their ramifications were in a healthy condition.

Case 3d.—The case of Carter resembled closely that of Shoemaker, with the exception that the larger bronchi were found diseased.

Case 4th.—Private Duncan was received into the Hospital on the 1st of May. For some time previous to his admission he had been affected with pneumonia, from which he was convalescing. On the morning of his admission he was seized with a chill, followed by fever, and in the evening the erysipelatous blush made its appearance on the face. At this time I ausculted the chest but could discern no implication as yet. On the third, the mouth became dry and parched, and the tongue was covered with a dirty leaden colored coat. Rhoncus and sibilus could be detected throughout the lungs. The difficulty of breathing was so great, that he could not maintain his position in bed for a moment; he was constantly tossing about and was unable to swallow even liquid. On the night of the 4th I ausculted his lungs and found crepitus throughout. He died on the 5th. The mucous membrane of the air passages was thickened, inflamed, and eroded. There was a good deal of serous effusion into the cellular tissue beneath the mucous membrane of the larynx and trachea, and the air cells and smaller bronchi were filled with a sero-purulent fluid.

Case 5th.—The case of Haley differed in no essential respect from that of Duncan. In each case the erysipelatous affection of the face existed a number of hours before the lungs were in the least affected, and the inflammation spread from the face to the mouth, and fauces, and from thence to the air passages. In the case of Shoemaker, death ensued before the inflammation had reached the bifurcation of the trachea, and was evidently owing to the closure of the glottis by the effusion of fluid beneath the mucous lining.

The treatment of the foregoing cases was such as the indications called for. I endeavored, but fruitlessly, to arrest the inflammation as soon as it involved the mouth, by a solution of nit. argenti. xx grs. to the ounce of water. As soon as the mucous tissue of the air passages was affected, the patients were kept nauseated by antimony, and such of them as lived through the "dry stage" were given carb. ammonia and other stimulating expectorants to assist them in expectorating. The vital energies were at too low an ebb to allow of my pushing the treatment by antimony to the extent that I desired. Tracheotomy was performed in two of the cases, but without any amelioration of the symptoms, other than slightly relieving the dyspnœa. Local depletion by means of cups applied to the chest and neck were resorted to, but without any beneficial results. The disease steadily advanced from the face to the mouth, and from thence to the air passages, destroying the patients in spite of my attempts to arrest it.

*Fort Grahem, Texas.*

---

To the Editors of the Medical Examiner:

Gentlemen:—I beg leave to enclose an interesting letter from Dr. A. Hays, in reference to the treatment of fracture of the os femoris. The modification of the long splint of Dessault, introduced by Dr. H., must prove exceedingly useful in many cases of injured thigh.

I am respectfully yours, Thos. D. Mütter.

*Philadelphia, Feb.* 1, 1849.

Dr. Mütter:

*Dear Sir:*—In a conversation I had with you on the subject of compound fractures of the lower extremities, particularly such as have been caused by gun-shot wounds, I mentioned to you that after using different dressings, I had found Dessault's, with Physick and Hutchinson's improvements liable to a difficulty in changing the dressings as frequently as necessary in such cases. The extension and counter extension being removed, a displacement took place in the ends of the fractured bones, and the renewal of it caused great pain and consequent fever and constitutional disturbance.

Under these circumstances, whilst employed as hospital surgeon on the northern frontier, in the war of 1812, I made use of Physick's improved splint, cutting out a portion of it opposite the wound, sufficiently large to allow free access to it, so as to dress it as often as necessary, without disturbing the extension and counter-extension. The two pieces of the long splint were united by a strong strip of iron, secured by screws; and it was made of such shape as to support the covering of the bed, and to keep it off the wounded part.

A thin piece of board was put between the portion of the limb dressed, and the ends of the splint, so as to give support to the part whence the segment of the splint had been removed.

This plan I found to meet my wishes and expectations very fully. The extension and counter-extension being continued, the dressing might be repeated as often as requisite without in the least disturbing the position of the limb.

The first case in which I adopted this plan was an officer near Plattsburg, New York, whose os femoris had been broken by a ball. Other dressings had been used, but he suffered so much constitutional disturbance by the frequent dressing necessary, that it was evident he must soon suffer amputation or sink into hectic fever. After the change in the dressing he improved rapidly.

I have no doubt the change in the long splint, as mentioned, will contribute very essentially to the cure of compound fractures of the lower extremeties.

Please excuse this hasty statement.

Yours respectfully,

A. Hays.

## Figures and Tables

**Figure f1:**